# Complex I-Associated Hydrogen Peroxide Production Is Decreased and Electron Transport Chain Enzyme Activities Are Altered in n-3 Enriched *fat-1* Mice

**DOI:** 10.1371/journal.pone.0012696

**Published:** 2010-09-13

**Authors:** Kevork Hagopian, Kristina L. Weber, Darren T. Hwee, Alison L. Van Eenennaam, Guillermo López-Lluch, José M. Villalba, Isabel Burón, Plácido Navas, J. Bruce German, Steven M. Watkins, Yana Chen, Alfreda Wei, Roger B. McDonald, Jon J. Ramsey

**Affiliations:** 1 VM Molecular Biosciences, University of California Davis, Davis, California, United States of America; 2 Department of Animal Science, University of California Davis, Davis, California, United States of America; 3 Department of Neurobiology, Physiology and Behavior, University of California Davis, Davis, California, United States of America; 4 Centro Andaluz de Biología del Desarrollo, Universidad Pablo de Olavide-CSIC, CIBERER, Instituto de Salud Carlos III, Sevilla, Spain; 5 Departamento de Biología Celular, Fisiología e Immunología, Universidad de Córdoba, Córdoba, Spain; 6 Department of Food Science and Technology, University of California Davis, Davis, California, United States of America; 7 Lipomics Technologies, West Sacramento, California, United States of America; 8 Department of Nutrition, University of California Davis, Davis, California, United States of America; Hospital 12 Octubre Madrid, Spain

## Abstract

The polyunsaturated nature of n-3 fatty acids makes them prone to oxidative damage. However, it is not clear if n-3 fatty acids are simply a passive site for oxidative attack or if they also modulate mitochondrial reactive oxygen species (ROS) production. The present study used *fat-1* transgenic mice, that are capable of synthesizing n-3 fatty acids, to investigate the influence of increases in n-3 fatty acids and resultant decreases in the n-6∶n-3 ratio on liver mitochondrial H_2_O_2_ production and electron transport chain (ETC) activity. There was an increase in n-3 fatty acids and a decrease in the n-6∶n-3 ratio in liver mitochondria from the *fat-1* compared to control mice. This change was largely due to alterations in the fatty acid composition of phosphatidylcholine and phosphatidylethanolamine, with only a small percentage of fatty acids in cardiolipin being altered in the *fat-1* animals. The lipid changes in the *fat-1* mice were associated with a decrease (*p*<0.05) in the activity of ETC complex I and increases (*p*<0.05) in the activities of complexes III and IV. Mitochondrial H_2_O_2_ production with either succinate or succinate/glutamate/malate substrates was also decreased (*p*<0.05) in the *fat-1* mice. This change in H_2_O_2_ production was due to a decrease in ROS production from ETC complex I in the *fat-1* animals. These results indicate that the fatty acid changes in *fat-1* liver mitochondria may at least partially oppose oxidative stress by limiting ROS production from ETC complex I.

## Introduction

Considerable interest exists in the possible health benefits of increasing dietary intake of n-3 fatty acids. It has been reported that diets high in n-3 fatty acids can decrease the incidence of several disease processes, including coronary heart disease [1], [2], inflammatory disorders [3], hypertension, and arthritis [1], as well as some mood disorders [4]. Nonetheless, some have raised questions concerning possible ill effects of consuming high levels of these nutrients. In particular, the polyunsaturated nature of n-3 fatty acids makes them susceptible to oxidative damage and it has been reported that supplementing rat diets with docosahexanoic acid (DHA; 22∶6 n-3) increases lipid peroxidation in the liver and kidneys [5]. Moreover, long-lived mammalian species have relatively high n-6∶n-3 ratios [6] and low tissue DHA levels [7], [8] compared to shorter-lived mammals. The inverse correlation between lifespan and n-6∶n-3 ratio is thought to reflect a greater potential for lipid peroxidation in the short-lived animals as a result of higher concentrations of n-3 polyunsaturated fatty acids (PUFA) [9]. However, several factors, including rate of reactive oxygen species (ROS) production and levels of antioxidants can influence oxidative stress, and the lipid environment around membrane proteins which produce ROS may be particularly important in determining the physiological response to n-3 fatty acids (or changes in the n-6∶n-3 ratio).

The mitochondrial inner membrane contains the enzyme complexes of the electron transport chain (ETC). These enzymes play a central role in energy metabolism and complexes I and III are sites of cellular ROS production [10]. Thus, the physiological response to alterations in membrane lipid composition would at least partially depend on the ability of the lipids to alter either the activity of these enzymes or the capacity of these enzymes to leak electrons and generate ROS. It has been clearly shown that the activities of the ETC enzymes are dependent on specific membrane phospholipids [11]. In particular, cardiolipin (CL) is required for activity of complexes I [12], III [12], [13], and IV [14], [15]. ETC complexes also bind phosphatidylcholine (PC) and phosphatidylethanolamine (PE), and it has been shown that these phospholipids are required for the optimum activity of complexes I and III [11]. While this requirement for phospholipids has been established, the influence of membrane n-3 fatty acids on ETC enzyme activities and ROS production is still open to debate. It has been shown that Complex IV activity is altered by diets that differ in n-3 fatty acid composition [16], but little is known about the influence of n-3 fatty acids on the activities of other ETC enzymes. The few studies that have investigated the influence of n-3 fatty acids on mitochondrial ROS production have been inconsistent, reporting either decreases [17] or increases [18], [19]. Additional studies are needed to gain a more complete understanding of the role n-3 fatty acids play in modulating mitochondrial enzyme activities and ROS production.

The recent availability of transgenic *fat-1* mice [20] has provided a unique genetic model for investigating the role that chronic alterations in n-3 fatty acids may play in mitochondrial ETC activity and ROS production. These mice express the *fat-1* gene from *C. elegans*, which encodes a desaturase that uses n-6 fatty acids as a substrate for the formation of n-3 fatty acids [21]. Transgenic *fat-1* mice express this gene ubiquitously and thus provide a model to investigate the effect of increasing tissue n-3 fatty acid levels without the need for dietary intervention to achieve this goal. This helps avoid the challenge of developing diets that truly differ only in the specific fatty acids of interest. The *fat-1* mice provide a good model for investigating the specific consequences of sustained increases in formation of n-3 fatty acids from n-6 fatty acid substrates.

The purpose of this study was to use *fat-1* mice to investigate the influence of increased n-3 fatty acid levels (and the resultant decrease in the n-6∶n-3 fatty acid ratio) on mitochondrial ROS production and ETC enzyme activities. Since previous studies have only measured tissue fatty acids in the *fat-1* mice, this study also determined the influence of the *fat-1* gene on the fatty acid composition and relative abundance of classes of mitochondrial phospholipids.

## Results

### Lipid Analysis

Lipids from Percoll-purified liver mitochondria were separated into five phospholipid classes: phosphatidylcholine (PC), phosphatidylethanolamine (PE), cardiolipin (CL), phosphatidylserine (PS), and lysophosphatidylcholine (LYS). There were no significant differences in the relative amounts (percent of total phospholipids) of these phospholipids from control and *fat-1* mitochondria (Table S1).

The fatty acid compositions associated with each phospholipid class are presented in Tables 1 and 2. PC, accounting for approximately 40% of mitochondrial phospholipids, exhibited significant decreases in percent PUFA (*p*<0.05) and n-6 (*p*<0.001) and significant increases in percent n-3 (*p*<0.001), monounsatruated fatty acids (MUFA) (*p*<0.01), and n-9 (*p*<0.01) in liver mitochondria from *fat-1* compared to control mice. Significant increases (*p*<0.05) in n-3 and decreases in n-6 fatty acids of PE (approximately 30% of mitochondrial phospholipids) and PS (4% of mitochondrial phospholipids) were also observed in the mitochondria from the *fat-1* mice. These differences account for an overall decrease (*p*<0.05) in the total n-6 to n-3 ratio. No differences (p>0.05) between the groups of mice were observed for any of the fatty acid series in CL.

**Table 1:** The ratios of n-6 to n-3 fatty acids and unsaturated to saturated fatty acids in liver mitochondria from control and fat-1 mice.

Phospholipid Class	Mice	n-6/n-3	Unsat/Sat^b^
Phosphatidylcholine	Control (n = 8)	2.21±0.07	1.46±0.03
	*fat-1* (n = 7)	1.52±0.06^a^	1.52±0.04
Phosphatidylethanolamine	Control (n = 8)	1.74±0.06	1.49±0.02
	*fat-1* (n = 7)	1.17±0.05^a^	1.51±0.04
Cardiolipin	Control (n = 6)	8.19±0.44	6.79±0.37
	*fat-1* (n = 5)	8.00±1.01	7.48±0.53
Phosphatidylserine	Control (n = 7)	0.88±0.05	0.99±0.03
	*fat-1* (n = 6)	0.67±0.11	1.03±0.06
Lysophosphatidylcholine	Control (n = 5)	2.80±0.21	0.75±0.02
	*fat-1* (n = 5)	1.87±0.27^a^	0.69±0.03

a Indicates a significant difference (*P*<0.05) between control and *fat-1* mitochondria within a specific phospholipid class.

b Unsat/Sat is the ratio of unsaturated to saturated fatty acids.

**Table 2:** The fatty acid composition of liver mitochondrial phospholipids from control and fat-1 mice.

Phospholipids^a^	Mice^b^	%SFA^a^	%MUFA^a^	%PUFA^a^	%n3	%n6	%n7	%n9
PC	Control (8)	40.6±0.5	11.1±0.5	48.2±0.4	15.0±0.2	33.2±0.5	2.8±0.2	8.5±0.4
	*fat-1* (7)	39.8±0.7	13.9±0.6^c^	46.3±0.6^c^	18.4±0.6^c^	27.8±0.5^c^	3.9±0.5	10.1±0.2^c^
PE	Control (8)	40.1±0.3	8.3±0.2	51.6±0.1	18.8±0.4	32.7±0.4	1.5±0.1	6.9±0.2
	*fat-1* (7)	39.9±0.6	9.1±0.5	51.0±0.3	23.4±0.6^c^	27.2±0.4^c^	2.1±0.3	7.3±0.4
CL	Control (6)	13.0±0.6	19.0±1.0	68.0±1.3	7.5±0.4	60.5±1.3	8.5±0.3	10.5±0.8
	*fat-1* (5)	12.0±0.8	20.0±1.2	68.0±1.3	7.8±0.7	60.1±1.8	8.4±0.7	11.6±0.5
PS	Control (7)	50.3±0.7	5.4±0.7	44.3±0.6	23.7±0.9	20.6±0.5	1.1±0.1	4.3±0.2
	*fat-1* (6)	49.4±1.4	12.3±3.1	38.3±2.5	23.5±2.4	14.7±0.9^c^	2.4±0.3^c^	9.9±2.8
LYS	Control (5)	57.3±0.7	11.1±0.5	31.7±0.5	8.4±0.5	23.2±0.7	3.3±0.4	7.7±0.2
	*fat-1* (5)	59.3±1.2	12.1±0.6	28.5±1.6	10.0±0.4^c^	18.4±1.8	3.3±0.3	8.5±0.3^c^

All values are expressed as a percent of total fatty acids within a phospholipid class.

a Abbreviations: PC, phosphatidylcholine; PE, phosphatidylethanolamine; CL, cardiolipin; PS, phosphatidylserine; LYS, lysophophatidylserine; %SFA, percent saturated fatty acids; % MUFA, percent monounsaturated fatty acids; %PUFA, percent polyunsaturated fatty acids.

b number in parenthesis indicates the number of animals used.

c Indicates a significant difference (*P*<0.05) between control and *fat-1* mitochondria within a specific phospholipid class.

There were a multitude of changes in the relative abundance of individual fatty acids in the different phospholipid classes as a result of *fat-1* gene expression (Tables S2-S6). Significant (*p*<0.05) decreases in arachidonic acid (20∶4 n-6) and increase in eicosapentaenoic acid (20∶5 n-3) were observed in all phospholipid classes from *fat-1* mice. Similarly, increases (*p*<0.05) in eicosatetraenoic acid (20∶4 n-3) occurred in all lipid classes from fat-1 mitochondria, except for PS. Increases in the n-3 fatty acids docosapentaenoic acid (22∶5 n-3) and DHA (22∶6 n-3) were also observed in mitochondrial PC and PE from the *fat-1* animals.

### Coenzyme Q levels

Coenzyme Q is a critical component of the mitochondrial electron transport chain and it can also acts as an antioxidant in the mitochondrial membrane. To determine if *fat-1* expression influences mitochondrial function through changes in coenzyme Q, levels of Q9, Q10 and total Q were measured in liver mitochondria (Table 3). There were no differences (*P*>0.10) between the control and *fat-1* mice for Q9, Q10, total Q or Q9/Q10.

**Table 3:** Coenzyme Q (CoQ) levels in liver mitochondria from control and fat-1 mice.

Mice^a^	CoQ9^b^	CoQ10^b^	Total CoQ^b^	CoQ9/CoQ10
Control (6)	977.8±91.4	206.3±77.0	1184.1±123.9	5.3±2.0
*fat-1* (6)	1076.3±136.5	207.2± 67.8	1283.4±194.8	5.5±1.3

a number in parenthesis indicates the number of animals used.

b values are expressed as pmol/mg protein.

### Electron transport chain enzyme activities

**Figure 1:**
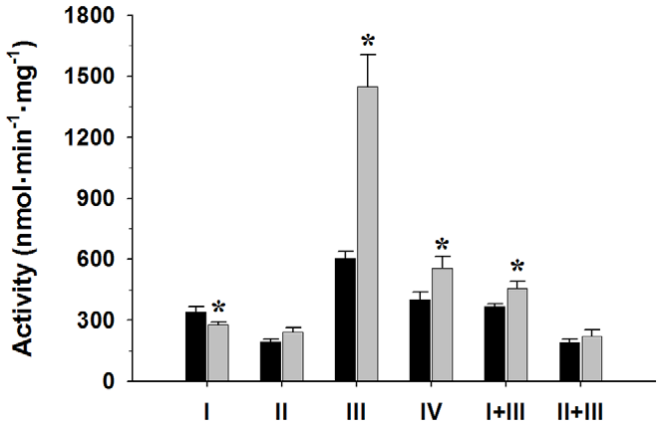
Activities of electron transport chain complexes in liver mitochondria from control (black bars, n = 9) and *fat-1* (grey bars, n = 7) mice. All measurements were completed at 30°C and activities are expressed nmol·min^−1^·mg mitochondrial protein^−1^. *Indicates a significant difference (*P*<0.05) between control and *fat-1* groups. Data are presented as means ± SEM.

Enzyme activities of the four mitochondrial ETC complexes, Complexes I – IV (Figure 1) were analyzed. To investigate a system which also includes coenzyme Q, we also measured Complex I+III and Complex II+III activities. The activity of Complex I was decreased by 19% (*p*<0.05) in the *fat-1* compared to control mice. In contrast, the activities of Complex III and Complex IV were increased by 58% (*p*<0.01) and 27% (*p*<0.05), respectively, in liver mitochondria from the *fat-1* mice. The decreased Complex I activity was not sufficient to cause an overall decrease in Complex I+III activity, and in fact there was a 19% increase (*p*<0.05) in Complex I+III activity in the *fat-1* animals. The activities of Complex II and Complex II+III were not significantly different between control and *fat-1* mice. The activities of Complex I+III and II+III were lower than the activity of Complex III alone. This result is expected since these assays require electrons to be transferred from Complex I (NADH) or Complex II (succinate) to coenzyme Q and Complex III. Thus, flux through Complex I or Complex II can constrain the activity of Complex III. This may reflect the fact that Complexes I and II are present at lower concentrations than Complex III in the mitochondrial membrane [22], [23], and coupling Complex III to other enzymes may blunt the “excess” capacity of this enzyme.

### H_2_O_2_ Production

**Figure 2:**
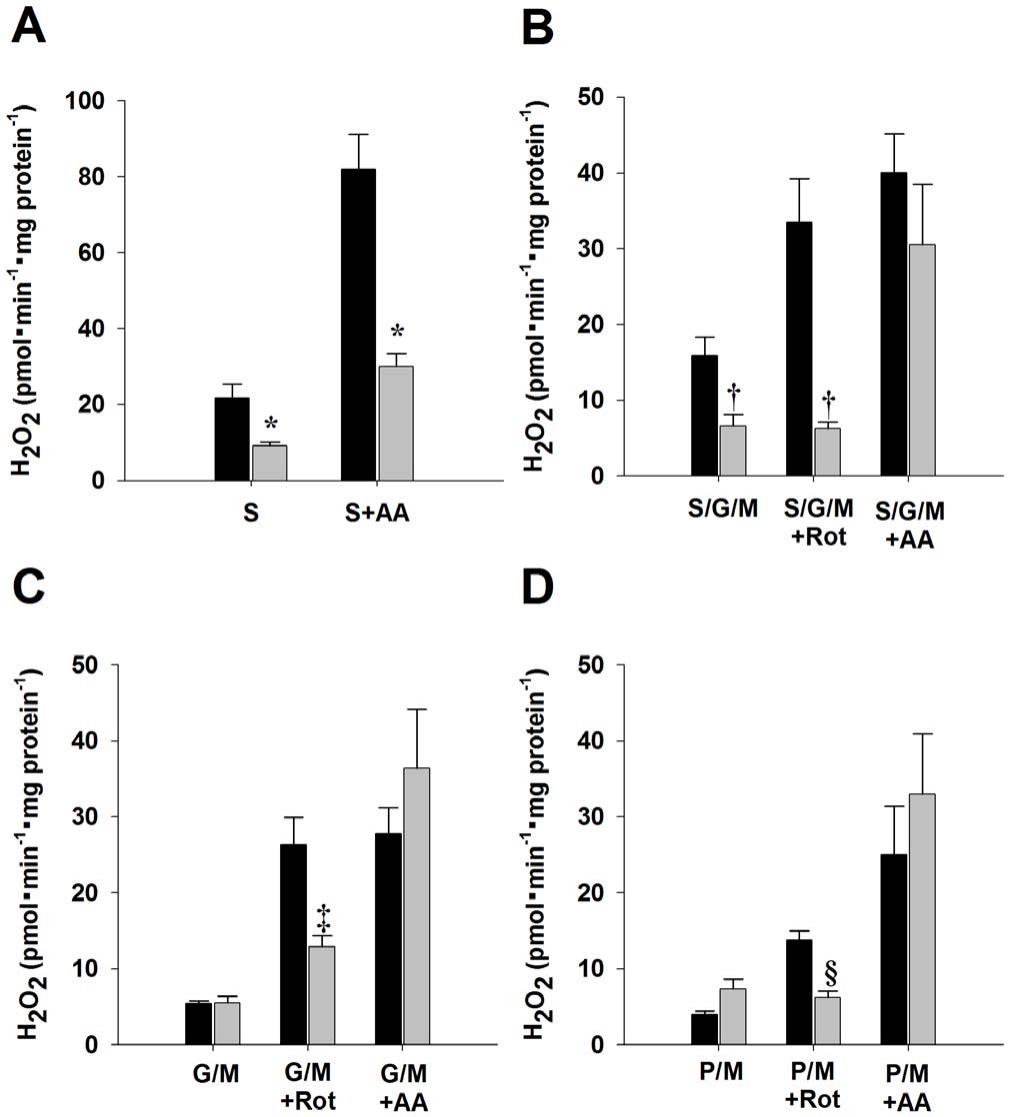
Hydrogen peroxide production in liver mitochondria from control (black bars, n = 7) and *fat-1* (grey bars, n = 5) mice. All measurements were completed on freshly isolated mitochondria. H_2_O_2_ production was monitored in mitochondria respiring on succinate (A), succinate/glutamate/malate (B), glutamate/malate (C), or pyruvate/malate (D). S, succinate; S/G/M, succinate/glutamate/malate; G/m, glutamate/malate; P/M, pyruvate malate; Rot, rotenone; AA, antimycin a. *, †, ‡, § indicates significant differences (*P*<0.05) between control and *fat-1* mitochondria respiring on succinate, succinate/glutamate/malate, glutamate/malate and pyruvate/malate, respectively. Data are presented as means ± SEM.

Hydrogen peroxide production was measured in mitochondria respiring on Complex I (pyruvate/malate, glutamate/malate), Complex II (succinate) or Complex I+II (succinate/glutamate/malate) linked substrates (Figure 2). In addition, inhibitors of Complex I (rotenone) and Complex III (antimycin A) were used to dissect sites of ROS production. The inhibitors maintain the electron transport chain in a reduced state on the substrate side of the inhibition. Therefore, if an inhibitor increases ROS production, the site of ROS generation must be on the substrate side of the inhibition. Under substrate-only conditions, a significant decrease was observed in *fat-1* H_2_O_2_ production in mitochondria respiring on succinate (*p*<0.05) and succinate/glutamate/malate (*p*<0.05). After addition of rotenone, *fat-1* mitochondria respiring on succinate/glutamate/malate, glutamate/malate or pyruvate/malate produced significantly less H_2_O_2_ when compared to controls (*p*<0.01). After addition of antimycin a, *fat-1* H_2_O_2_ production was significantly decreased when succinate was the substrate (*p<*0.001). However, with all other substrates, no significant differences in H_2_O_2_ production following antimycin a addition were observed between liver mitochondria from *fat-1* and control mice. The results indicate that H_2_O_2_ production was greatly decreased in *fat-1* liver mitochondria under conditions of maximum ROS production from complex I by forward (rotenone with complex I linked substrates) or reverse (succinate or succinate with antimycin A) electron flow.

### Proton Leak Kinetics

**Figure 3:**
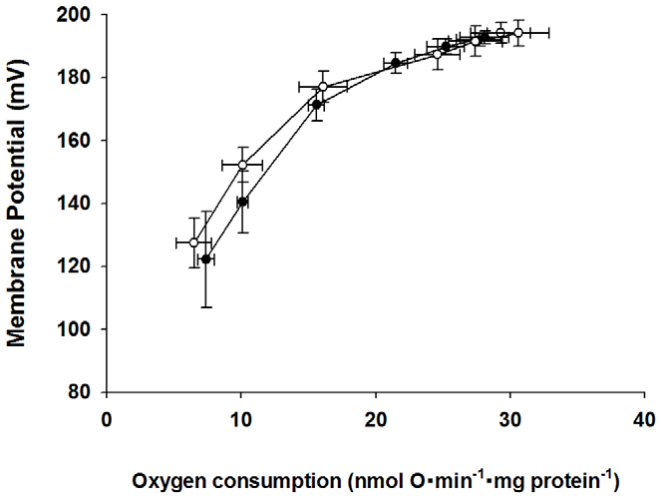
Liver mitochondrial proton leak kinetics in control (filled circles, n = 6) and *fat-1* (open circles, n = 6) mice. Proton leak kinetics were completed with 10 mM succinate and 8 µg/mg protein oligomycin and were titrated with 0.1–2.4 mM malonate. Conditions used for these measurements are described in the text. Data are presented as means ± SEM.

Mitochondrial proton leak is a major stimulator of mitochondrial respiration, and consequently this process can also influence mitochondrial ROS production. To determine if the lipid changes occurring with *fat-1* expression influence mitochondrial proton leak, liver mitochondrial proton leak kinetics were determined for the *fat-1* and control mice (Figure 3). There were no differences in maximal leak-dependent respiration and membrane potential (points farthest to the right in the graph) between the two groups of mice. No differences in the proton leak kinetics plots were observed between the *fat-1* and control mice, indicating that basal proton leak was not altered in liver mitochondria from these animals.

### Lipid Peroxidation

**Figure 4:**
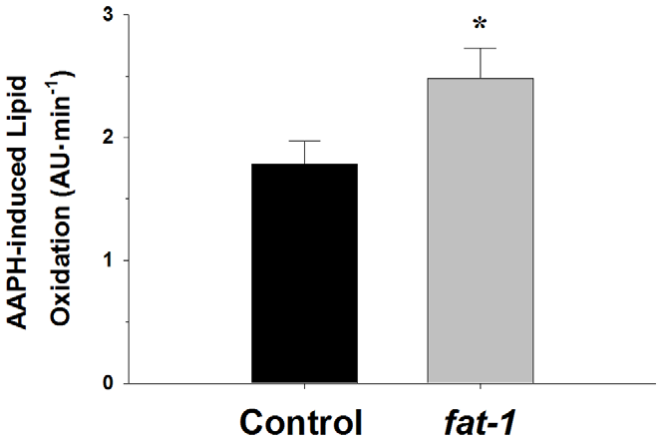
Susceptibility to 2,2′-azobis(2-amidinopropane) (AAPH)-induced lipid peroxidation in liver mitochondria from control and *fat-1* mice. AAPH-induced lipid peroxidation was determined by measuring the loss in fluorescence of *cis*-parinaric acid (*c*PN) incorporated into mitochondrial membranes. Peroxidation was initiated by AAPH at 37°C. Values are presented as arbitrary fluorescence units per minute for *c*PN. * indicates a significant difference (*P*<0.05) between the control and *fat-1* groups. Data are presented as means ± SEM.

**Figure 5:**
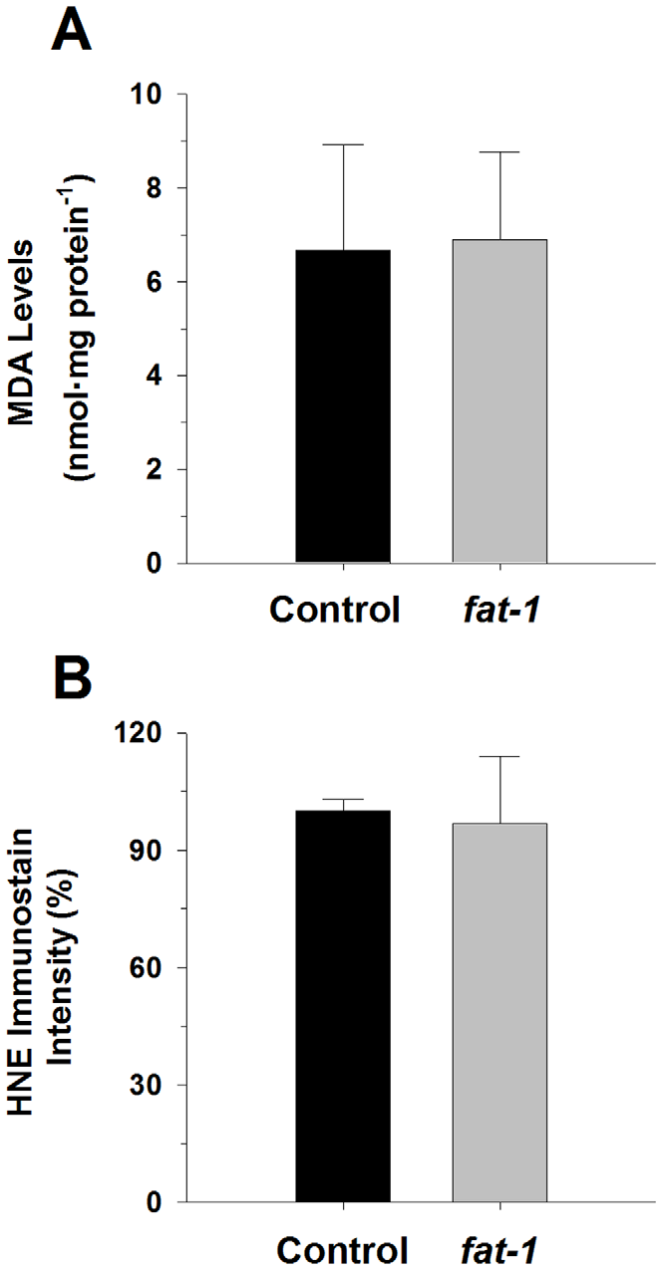
Basal lipid peroxidation in liver mitochondria from control and *fat-1* mice. A) Lipid peroxidation was determined by measuring the level of malondialdehyde (MDA) in mitochondria as described in the text. There were no differences (*P*>0.05) between the control and *fat-1* groups. B) Immunochemical detection of 4-hydroxynonenal (HNE) adducts in isolated mitochondria. HNE measurements were completed as described in the text. Relative densitometry values expressed as means of four independent samples from each group. The immunostain intensity of the control group was assigned a value of 100 percent. There were no differences (*P*>0.05) between groups. Data are presented as means ± SEM.

Susceptibility of mitochondrial membranes to lipid peroxidation was assessed by measuring loss of *cis*-parinaric acid (cPN) fluorescence in response to 2,2′-azobis(2-amidinopropane) (AAPH) (Figure 4). A significant increase (*P*<0.05) in mitochondrial membrane lipid peroxidation was observed in the *fat-1* mice following stimulation of peroxidation with AAPH. This result indicates that the increase in membrane n-3 fatty acids in the *fat-1* mice is associated with an increase in susceptibility to peroxidation when faced with an oxidative insult. It was necessary next to determine if alterations in mitochondrial lipid peroxidation occurred in the *fat-1* animals under basal conditions. Two methods, malondialdehyde (MDA) and 4-hydroxynonenal (HNE), were also used to provide an indication of basal levels of lipid peroxidation in mitochondria from the *fat-1* mice (Figure 5A and 5B). In contrast to the AAPH results, no differences (*P*>0.05) in MDA or HNE levels were observed in mitochondria from the two groups of mice. These results indicate that despite elevated n-3 levels, basal lipid peroxidation is not increased in mitochondria from 1 year old *fat-1* mice.

### β-oxidation

Oxidation of palmitic acid was measured as an indicator of the rate of β-oxidation (Figure S1). There were no differences (*P*>0.05) between the two groups of mice for palmitate oxidation.

## Discussion

The purpose of this study was to use transgenic *fat-1* mice to investigate the influence of increased n-3 fatty acid levels on mitochondrial ROS production and ETC enzyme activity. To determine if the *fat-1* mouse was an appropriate model to use for these studies, it was first necessary to determine the mitochondrial lipid composition of these animals. While several studies have reported the influence of the *fat-1* gene on tissue fatty acid composition and the n-6∶n-3 fatty acid ratio [24], [25], we are not aware of any studies that have measured lipid composition in mitochondria isolated from these mice. The results of this study showed that liver mitochondria mirrored the pattern of fatty acid changes observed in whole liver from *fat-1* mice [20], [26].

Similar to previous studies investigating the influence of *fat-1* gene expression on tissue fatty acids [20], [26], mitochondria from the *fat-1* mice showed an increase in n-3 fatty acids and a decrease in the ratio of n-6∶n-3 fatty acids. This change in the ratio of n-6∶n-3 fatty acids was entirely attributable to fatty acid alterations in PE and PC, which together accounted for greater than 70% of liver mitochondrial phospholipids. Changes were limited to n-6 and n-3 fatty acids of 20 carbons in length, or greater. Measurements of tissue fatty acids in liver [26] have also shown that changes in the n-6∶n-3 ratio are due to alterations in fatty acids of 20 carbons or longer with no significant changes in linoleic acid. The *fat-1* gene encodes a desaturase which has been shown in *Arabidopsis thaliana* to synthesize n-3 fatty acids from 18 or 20 carbon n-6 substrates [21]. However, the fact that linoleic acid (C18∶2 n-6) is not decreased in either liver mitochondria or whole tissue [26] indicates that 20 carbon n-6 fatty acids, such as arachidonic acid (C20∶4 n-6), are likely the primary substrates for n-3 fatty acid synthesis.

It is interesting to note that *fat-1* animals had only minor changes in the fatty acid composition of CL, and that the few significant changes were limited to fatty acids that constituted less than 5% of the total fatty acids in this phospholipid. It has been reported that the fatty acid composition of CL is extremely sensitive to changes in dietary fatty acids [27]. In particular, studies in rats have shown that feeding fish oil causes an increase in DHA and a decrease in linoleic acid levels in CL from heart [28], [29] and liver [29]. These studies revealed a disparate response in CL fatty acid composition as compared to what was observed in the present study. The reason for these differences may reflect the fact that the dietary-manipulation studies produced very large changes in mitochondrial fatty acid composition. In contrast, the *fat-1* and control mice in the present study were fed the same diet and the magnitude of the mitochondrial lipid changes were much smaller than those reported in many of the dietary manipulation studies. Also, studies feeding fish oil [28], [29] had a significant decrease in diet and mitochondrial linoleic acid levels while the levels of this fatty acid were not altered in the *fat-1* mice. This is of importance, since linoleic acid is the primary fatty acid in liver mitochondria from rats and mice fed a wide range of diets [30]. The *fat-1* mouse provides a unique model for investigating changes in the n-3 fatty acid composition of liver mitochondria that are independent of alterations in either the amount or the fatty acid composition of CL. The results from the fat-1 mice also demonstrate that major alterations in mitochondrial ROS production and ETC enzyme activities can occur with fatty acids changes in PE and PC, and without major fatty acid changes in CL.

It is important to note that expression of the *fat-1* gene, or any foreign gene, in mice clearly does not reflect a normal physiological condition. The results of our study show that the fatty acid changes which occur in the *fat-1* mouse do not entirely mimic those observed with dietary fatty acid alterations. Thus, the *fat-1* mouse and dietary lipid manipulations should be viewed as distinct tools for manipulating membrane lipid composition. Future studies using dietary lipid manipulations in *fat-1* mice may prove useful in helping to tease out the role of specific fatty acid in mitochondrial function.

A few studies have used dietary fish oil to investigate the influence of n-3 fatty acids on mitochondrial respiration [29], [31], [32], [33], [34], [35], and these studies have reported either no change [32], [34], [35], decreases in some (but not all) measures using various substrates [29], [33] or increases in succinate supported respiration [31]. However, measures of mitochondrial respiration do not necessarily reflect changes in maximum mitochondrial ETC activity and do not provide information about alterations in the activity of specific ETC enzymes. It has been reported that there is excess capacity of ETC enzymes and that large changes in maximum ETC enzyme activities may be needed before respiration is altered [22], [36]. For this reason, it is essential to measure the activity of individual ETC enzyme complexes to truly determine the influence of n-3 fatty acids on the ETC. There have been a couple of studies that have looked at the influence of n-3 fatty acids and changes in the ratio of n-6∶n-3 fatty acids on the activities of specific mitochondrial ETC enzyme complexes in liver [16], [29], and none of these studies have measured the activities of all of four complexes (I, II, III and IV). It has been reported [16] that liver cytochrome oxidase (Complex IV) activity in rats is increased with short-term (25 weeks) and decreased with long-term (60 weeks) consumption of fish oil. It has also been [29] reported that Complex IV activity is decreased in heart mitochondria from rats consuming a fish oil containing diet for 30 days. These fish oil diet studies provided the animals a rather extreme diet that contains very high levels of n-3 fatty acids, which resulted in highly elevated levels of n-3 fatty acids as compared to those observed in the *fat-1* animals. A recent study reported that lipid peroxidation leads to a decrease in the activities of ETC complexes III and IV [37], and it is possible that the decreases in complex IV activity in previous studies was due to lipid peroxidation secondary to very large increases in membrane unsaturation. The present study was unique in that it looked at the activities of each of the ETC enzyme complexes, and did not focus on a single enzyme. This was important since the results showed that the fatty acid changes in the *fat-1* mice did not cause uniform changes in the activities of ETC enzymes. It has been reported that increases in the ratio of complex IV to complex I enzyme activities are associated with a decrease in ROS production and oxidative damage [38], [39], possibly by stimulating electron flux through complex I. Our results are consistent with this conclusion and suggest that the changes in the activities of the ETC complexes observed in the *fat-1* mice may help limit ROS production. In particular, increases in the activities of complexes III and IV may help to stimulate electron flow through complex I and prevent this complex from remaining in a reduced state.

While many studies have focused on polyunsaturated fatty acids as sites of oxidative damage, there is much less information about the influence of n-3 fatty acids or alterations in the n-6∶n-3 ratio on mitochondrial ROS production. It has been reported that feeding fish oil increases ROS production in rat colonocytes [18] and heart mitochondria [40]. However, both of these studies appear to involve increases in ROS production that may be secondary to peroxidation of mitochondrial phospholipids. In contrast to these findings, H_2_O_2_ production was either unchanged or decreased in liver mitochondria from rats consuming fish oil when there was no increase in mitochondrial oxidative damage [17]. The results of the present study are consistent with this latter finding, and with the notion that liver mitochondrial n-3 fatty acids may lead to a decrease in ROS production. In particular, mitochondria from the *fat-1* mice had decreased H_2_O_2_ production (*P*<0.05) when respiring on succinate/glutamate/malate or succinate alone.

Mitochondrial H_2_O_2_ production was also decreased (*P*<0.05) with all substrates in these animals following the addition of rotenone. The ETC inhibitors rotenone (complex I) and antimycin a (complex III) were used to identify sites of mitochondrial ROS production. The ETC inhibitors increase the redox state of the electron carriers on the substrate side of the inhibitor and thus if a particular inhibitor increases ROS production, the site of ROS generation must be on the substrate side of the inhibitor [41]. The fact that rotenone did not greatly stimulate H_2_O_2_ production in *fat-1* mitochondria respiring on complex I-linked substrates indicates that liver complex I from these animals produced very little ROS. It has been reported that Complex I produces ROS from both the FMN and ubiquinone binding site [42], with the ubiquinone binding site being the location of ROS production during backflow of electrons from complex II into Complex I [43]. The fatty acid changes in the *fat-1* mice appear to decrease ROS production from both sites in Complex I. When antimycin a is added to mitochondria respiring on succinate (complex II-linked substrate) ROS production would be stimulated from both complex III and reverse electron flow into complex I. Under these conditions, there was a decrease (*P*<0.05) in H_2_O_2_ production from the *fat-1* mitochondria. This difference appears to be solely due to a decrease in ROS production from backflow into complex I, since no differences in H_2_O_2_ production were observed between control and *fat-1* mice when antimycin a was added to mitochondria respiring only on complex I-linked substrates. Taken together, these results indicate that the fatty acid changes which occur in the *fat-1* mouse decrease the ability of complex I to leak electrons and form ROS. This is particularly important since complex I appears to be a major site of mitochondrial ROS production [10]. Thus, this decrease in ROS production from complex I may serve as a protective mechanism against oxidative stress in membranes with increased levels of n-3 fatty acids.

The mechanism for decreased ROS production from complex I in liver mitochondria of the *fat-1* mice is not known. It has been reported that ROS production during reverse electron flow is highly dependent on mitochondrial proton motive force while during forward electron flow ROS production is primarily dependent on NADH/NAD [10]. Thus, it follows that mitochondrial proton leak could decrease ROS production by decreasing both NADH/NAD and mitochondrial proton motive force. A positive correlation between mitochondrial DHA levels and proton leak has been reported [44], therefore, the influence of fatty acid changes in the *fat-1* mice on basal proton leak was assessed in the present study. There was no change in basal proton leak in liver mitochondria from the *fat-1* and control mice, indicating that increases in basal proton leak were not responsible for the decrease in ROS production in the fat-1 mitochondria.

The mechanism responsible for decreased Complex I-linked ROS production in the *fat-1* mice could be due to either direct or indirect actions of n-3 fatty acids on mitochondria. It is thought that the formation of mitochondrial supercomplexes may limit ROS production from Complex I [23], and it is possible that alterations in n-3 fatty acids may influence supercomplex formation. Also, it has been shown that n-3 fatty acids play a role in regulating gene transcription[45], [46] and studies in adipocytes show that n-3 fatty acids stimulate mitochondrial biogenesis [47]. It is possible that n-3 fatty acids could influence ETC enzyme activities and ROS production in liver through alterations in the transcription of genes encoding ETC proteins. It is also important to consider that the results observed in liver could be influenced by *fat-1* expression in other tissues. Additional studies are needed, preferably using mice that express the *fat-1* gene under temporal and spatial control, to determine the mechanism responsible for the observed changes in ETC enzyme activity and ROS production in *fat-1* mice.

Expression of the *fat-1* desaturase results in elevated levels of polyunsaturated n-3 fatty acids in tissues and mitochondria, and this alone would be expected to increase tissue susceptibility to oxidative damage. However, no differences in markers of basal oxidative damage were observed in the present study between wild-type and *fat-1* mice at 1 year of age. This suggests that adaptations occurred to allow these mice to live with elevated n-3 fatty acid levels. It has recently been shown that the activities of catalase and superoxide dismutase are not significantly changed in liver from *fat-1* mice [48], suggesting that an upregulation of hepatic antioxidant enzymes is not an adaptation to *fat-1* expression. In contrast, the present study shows that *fat-1* expression modulates mitochondrial H_2_O_2_ production. In particular, there was a decrease in complex I-linked ROS production in the *fat-1* mice. It was shown that *fat-1* expression leads to alterations in the activities of mitochondrial ETC enzymes (the major site of mitochondrial ROS production), and these changes could influence energy metabolism and ROS production in the *fat-1* mice.

In conclusion, this is the first study to show that expression of the *fat-1*desaturase in mice, which increases n-3 fatty acids in tissues and mitochondria, leads to alterations in ETC enzyme activities and a dramatic decrease in ROS production from complex I of the ETC. This decrease in ROS production may serve as a protective mechanism against oxidative stress in membranes containing increased amounts of n-3 fatty acids.

## Materials and Methods

### Materials

General laboratory chemicals and substrates were purchased from Sigma Aldrich (St. Louis, MO), except Percoll (Pharmacia, Piscataway, NJ), bovine serum albumin (MP Biochemicals, Santa Ana, CA), and NADH (Roche, Indianapolis, IN).

### Animals

Male heterozygous *fat-1* transgenic mice were provided by Dr Jing Kang (Department of Medicine, Massachusetts General Hospital and Harvard Medical School) and used to establish a breeding colony at UC Davis, from which all mice were used in this study. The mice were group-housed (up to 4 mice per cage) and maintained in a temperature (22–24°C) and humidity (40–60%) controlled animal facility, with a 12 hour light:dark cycle. All animals were fed ad libitum a Purina 5008 rodent diet, with free access to water. The animal care and use protocol (#13138) was approved by the UC Davis Institutional Animal Care and Use Committee.

### Mitochondrial isolation

One year old mice were euthanized by cervical dislocation, and the liver removed immediately and weighed. All subsequent procedures were performed at 4°C. Mitochondria were isolated using the method of Venditti et al. [49] with some modifications. Briefly, liver tissue was washed and minced in ice cold isolation medium (220 mM mannitol, 70 mM sucrose, 20 mM Tris, 1 mM EDTA, 0.1% BSA, pH 7.4), homogenized (10% w/v in isolation medium) in an ice-cold glass-Teflon motor-driven homogenizer and centrifuged at 500 g for 10 min in a Beckman Coulter Model J2-21M Centrifuge. The pellet was discarded and the supernatant was centrifuged at 10,000 g for 10 min. The resulting pellet was re-suspended and centrifuged at 10,000 g in the above isolation medium and this step was repeated twice. The resulting mitochondria were washed twice with 0.15 M KCl to remove catalase. The final mitochondrial pellet was re-suspended in isolation medium without BSA, centrifuged as above and the resulting final pellet re-suspended in isolation medium without BSA and used in the experiments. A portion of this was used for hydrogen peroxide assays and proton leak, while another portion was used for ETC assays. The rest was further purified on Percoll gradients and the resulting mitochondria were separated and stored in liquid nitrogen for future lipid analysis.

### Percoll gradient purification of mitochondria for lipid analysis

To prepare mitochondria for lipid analysis, a discontinuous Percoll gradient [50] was used, containing equal volumes of Percoll at 18%, 30%, and 60% concentrations (v/v) in a buffer of 0.225 M mannitol, 1 mM EGTA, and 25 mM HEPES. Re-suspended mitochondria were loaded on the gradient and centrifuged at 8700 g for 1 hr at 4°C. After centrifugation, mitochondria were isolated from the 30–60% interface and the Percoll removed via two rounds of centrifugation at 10,000 g for 10 min in isolation media without BSA, as described previously. The purified mitochondria were re-suspended in isolation buffer and stored in liquid nitrogen until lipid analysis was performed.

### Electron transport chain (ETC) complex activities

The activities of the ETC complexes I, II, III and IV were assessed spectrophotometrically using a Perkin Elmer Lambda 25 UV/Vis Spectrometer [51], [52], [53], [54]. All assays were performed at 30°C using 25 mM potassium phosphate buffer, pH 7.2 (assay buffer), in a final assay volume of 1 mL. Complex I (NADH:ubiquinone oxidoreductase) activity was measured in the assay buffer to which 5 mM MgCl_2_, 0.13 mM NADH, 65 µM ubiquinone-1, 2 mM KCN, 2.5 mg/ml BSA, and 5 µg/ml antimycin a were added. The assay was started by the addition of mitochondria and the change in absorbance at 340 nm (ε = 6.22 mM^−1^cm^−1^) due to NADH oxidation in the presence of ubiquinone-1 was recorded for 1minute, after which 5 µg/ml rotenone was added and the absorbance recorded for a further 2 minutes to allow for the quantification of the rotenone-sensitive activity. Complex II (succinate:ubiquinone oxidoreductase) activity was determined by the change in absorbance at 600 nm (ε = 19.1mM^−1^cm^−1^) due to the reduction of 2,6-dichlorophenolindophenol (DCPIP). 20 mM succinate and mitochondria were added to the assay buffer, and incubated for 10 minutes at 30°C, followed by the addition of 5 mM MgCl_2_, 2 mM KCN, 5 µg/ml antimycin A, 5 µg/ml rotenone, 50 µM DCPIP and finally 65 µM ubiquinone-1 to start the assay. The reaction was monitored for a further 10 minutes. Complex III (ubiquinol:ferritocytochrome *c* oxidoreductase) activity was determined by the change in absorbance at 550 nm (ε = 19.1 mM^−1^cm^−1^) due to the reduction of oxidized ferritocytochrome *c*. To the assay buffer were added 5 mM MgCl_2_, 2 mM KCN, 5 µg/ml rotenone, 2.5 mg/ml BSA, 50 µM oxidized cytochrome c, and mitochondria. The assay was started by adding 60 µM decylubiqunol and absorbance recorded for one minute, taking the initial 30 seconds rate for calculation. The assay was also performed in the presence of 5 µg/ml antimycin a to distinguish between non-enzymic reduction of ferricytochrome c and that due to reduction by decylubiquinone. Decylubiquinol was prepared as described [55]. Complex IV (cytochrome *c* oxidase) activity was determined by the change in absorbance at 550 nm (ε = 19.1 mM^−1^cm^−1^) due to the oxidation of reduced cytochrome c. 15 µM reduced cytochrome c and mitochondria were added to the assay buffer and the reaction was followed for 2 minutes.

The activity of complex I+III (NADH:cytochrome c oxidoreductase) was measured at 30°C by the change in absorbance at 550 nm (ε = 19.1 mM^−1^cm^−1^) due to the reduction of oxidized ferricytochrome c. The assay final volume was 1 ml and contained 50 mM potassium phosphate buffer pH 7.4, 80 µM ferricytochrome c, 5 mM MgCl_2_, 100 µM NADH, 2 mM KCN and mitochondria were added to start the reaction. After 1 minute of reading, 5 µg/ml rotenone was added and the reaction followed for a further 2 minutes. Complex II+III (succinate:cytochrome *c* oxidoreductase) activity at 30°C was determined by the change in absorbance at 550 nm (ε = 19.1 mM^−1^cm^−1^) due to the reduction of ferricytochrome c. The assay final volume was 1 ml and contained 40 mM potassium phosphate buffer, 0.5 mM EDTA, 20 mM succinate and mitochondria. This was incubated for 10 minutes at 30°C, after which 2 mM KCN, 5 µg/ml rotenone and 30 µM ferricytochrome c was added to start the reaction.

### Mitochondrial Proton Leak Kinetics

Mitochondrial oxygen consumption was measured using previously described methods [49], [56]. Oxygen consumption was measured at 30°C using a Clark-type oxygen electrode (Hansatech, Norfolk, UK). All measurements were completed in duplicate using mitochondria (0.5 mg mitochondrial protein/ml) in incubation medium (145 mM KCl, 30 mM Hepes, 5 mM KH_2_PO_4_, 3 mM MgCl_2_, 0.1 mM EGTA, 5 µM rotenone, and 0.4 µg nigericin/mg mitochondrial protein, pH 7.4). Respiration was initiated by the addition of succinate (10 mM) without ADP (state 4) or in the presence of 500 µM ADP (state 3). Respiratory control ratio was calculated as state 3 divided state 4 respiration. This value was 4.0±0.3 and 3.8±0.2 for the *fat-1* and control mice, respectively. Mitochondrial membrane potential was assessed simultaneously with all measurements of oxygen consumption [56]. Membrane potential was measured with a methyltriphenylphosphonium (TPMP^+^) electrode using previously published methods [57]. The TPMP^+^ electrode was calibrated with sequential additions of 0.5 µM TPMP^+^ until a total concentration of 2.5 µM TPMP^+^ was achieved. A TPMP^+^ binding correction of 0.4 was used for membrane potential calculations [56]. Proton leak kinetics were determined by titrating the ETC with malonate (0.1–2.4 mM), an inhibitor of complex II, in the presence of oligomycin (8 µg/mg mitochondrial protein).

### Electrophoresis and Western Blotting

Liver mitochondrial proteins from *fat-1* and control mice were separated on 10% Bis-Tris SDS-polyacrylamide gels using the Invitrogen NuPAGE Electrophoresis System (Invitrogen Corporation, Carlsbad, CA) and MOPS running buffer. Proteins (3 µg per lane) were resolved for 60 minutes at 200 V and blotted onto nitrocellulose membranes at 20 V for 7.5 minutes using the Invitrogen iBlot Dry Blotting System (Invitrogen Corporation, Carlsbad, CA). Following blotting, the membranes were briefly rinsed in TBS before blocking in Odyssey Blocking Buffer (LI-COR Biosciences, Lincoln, Nebraska) for two hours at room temperature. After blocking, the membranes were probed overnight at 4°C with primary antibodies diluted in Odyssey Blocking Buffer. A polyclonal anti-serum to 4-hydroxynonenal (HNE) adducts was used at 1∶5,000 dilution (Alpha Diagnostic, San Antonio, TX) [58]. A monoclonal antibody to human porin (Molecular Probes, Eugene, OR) was used as a mitochondrial protein standard at 1∶15,000 dilution [59], [60]. After overnight incubation, the membranes were washed 4 times, 5 minutes each, with 0.1% TBST and then incubated for one hour at room temperature with secondary antibodies. IRDye 680 Goat Anti-Mouse and IRDye 800 Goat Anti-Rabbit fluorescent secondary antibodies (LI-COR Biosciences, Lincoln, Nebraska) were each used at a 1∶20,000 dilution. After incubation, the membranes were washed 4 times, 5 minutes each, with 0.1% TBST, and then briefly rinsed with TBS to remove traces of Tween-20. The membranes were visualized and relative quantities of the proteins analyzed using the Odyssey Infrared Imaging System and associated software (LI-COR Biosciences, Lincoln, Nebraska).

### H_2_O_2_ assays

Hydrogen peroxide production by liver mitochondria was determined fluorimetrically, as described previously [56], [61], at 37°C, using a Perkin Elmer LS55 luminescence spectrometer, with sample compartment equipped with stirring mechanism. The excitation and emission wavelengths of the luminescence spectrometer were 320 and 400 nm, respectively The assay buffer was 10 mM potassium phosphate pH 7.4, containing 154 mM KCl, 0.1 mM EGTA, 3 mM MgCl_2_, 500 µg/assay *p-*hydroxyphenylacetic acid (PHPA), 4 units of horseradish peroxidase, plus substrates as indicated below, in final volume of 3 ml. Substrates used included 10 mM pyruvate/5 mM malate (P/M), 5 mM glutamate/5 mM malate (G/M), 10 mM succinate (Suc), 10 mM succinate/5 mM malate (Suc/M), and 10 mM succinate/5 mM glutamate/5 mM malate (Suc/G/M), in the presence or absence of the inhibitors rotenone (complex I) and antimycin a (complex III). H_2_O_2_ levels were expressed as picomoles of H_2_O_2_ per minute per milligram of protein. A standard curve was generated over a range of H_2_O_2_ concentrations, and was used to convert the fluorescence readings from assays into H_2_O_2_ concentrations.

### Lipid Analysis

Lipid analysis was performed on Percoll-purified mitochondria by Lipomics Technologies (Tethys Biosciences, Sacramento, CA). Briefly, lipids were extracted from the mitochondria with chloroform-methanol [62]. Lipid classes were separated by preparative thin layer chromatography [63]. The lipid fractions were scraped from the thin layer plates and methylated with 3N methanolic hydrochloride under a nitrogen environment in a sealed vial maintained at 100°C for 45 minutes. The resulting fatty acid methyl esters were extracted with hexane containing 0.05% butylated hydroxytoluene. Fatty acid methyl esters were separated and quantified by capillary gas chromatography [63].

### Coenzyme Q Determination

Coenzyme Q levels were determined by HPLC as previously described [64]. Briefly, mitochondria were treated with 2% SDS followed by two volumes of ethanol:isopropanol (95∶5). Q was recovered from this solution by extraction with five volumes of hexane. After hexane evaporation, the lipid extract was re-suspended in ethanol, dried again by speed-vac, re-suspended in ethanol and analyzed by HPLC equipped with an electrochemical (ESA, Chelmsford, MA) detector (−500 mV/+300 mV). Separation was carried out on a C18 column (5-µm particle, 5X0.45 cm) with a mobile phase of n-propanol/methanol (35∶65) containing 13.9 mM lithium perchlorate, at a flow rate of 1 ml/min. Concentrations of CoQ9 and CoQ10 were calculated by integration of peak areas and comparison with external standards [65].

### AAPH-induced Lipid Peroxidation

Membranes were assayed for susceptibility to lipid peroxidation in an incubation medium consisting of 0.2 mM Tris-HCl buffer, pH 7.6, 150 µg mitochondrial membranes and 3 µM *cis*-parinaric acid (cPN) in a final volume of 2 ml. Peroxidation was initiated by thermal decomposition of 2 mM 2,2′-azobis(2-amidinopropane) (AAPH) at 37°C. The loss in fluorescence of *c*PN incorporated into mitochondrial membranes was employed as an indirect indicator of AAPH-induced lipid peroxidation [66]. Fluorescence was recorded continuously in an Aminco-Bowman Series 2 Luminiscence Spectrometer set at 324 nm for excitation (slit of 4 nm) and 413 nm for emission (slit of 8 nm). Peroxidation rates were expressed as the decay in fluorescence (Arbitrary Units) per min.

### Measurement of Mitochondrial MDA

Malondialdehyde (MDA) levels were measured according to the method of Gérard-Monnier et al. [67] with some modifications. Briefly, the reaction mixture contained 6.6 mM N-methyl-2-phenyl-indol (MPI), 0.015 mg/ml butylated hydroxytoluene and 5.55% HCl. The assay was initiated by the addition of sample (0.5–1 mg) and incubated at 45°C for 45 min. To determine the amount of MDA, known concentrations of 1,1,3,3-tetra-ethoxypropane (malondyaldehide bis(diethyl-acetal) (0–20 nmol) were used.

### Measurement of β-oxidation Activity

Palmitate oxidation was measured as an indicator of β-oxidation [68]. Briefly, the reaction mixture contained 50 ml Tris/HCl, pH 8.0, 40 mM NaCl, 2 mM KCl, 2 mM MgCl_2_, 1 mM DTT, 5 mM ATP, 0.2 mM L-carnitine, 0.2 mM NAD, 0.6 mM FAD, 0.12 mM CoA, 0.1 µCi [^14^C]palmitic acid, and 100 µg mitochondrial protein in a final volume of 200 µl. The reaction was initiated by adding substrate and incubated at 37°C for 20 minutes. The reaction was terminated by adding 200 µl of 0.6N perchloric acid followed by centrifugation. The resulting supernatant was extracted three times with 800 µl of N-hexane to remove any remaining palmitate and radioactivity of the aqueous phase was measured.

### Statistical Analysis

All variables were tested for normality using the Shapiro-Wilk's test. Comparisons between control and *fat-1* groups were completed using an unpaired Student's *t*-test. For sample groups that did not meet the requirements for normal distribution, comparisons between the control and *fat-1* groups were completed using the Wilcoxon's two-sample rank test. Respiration and membrane potential comparisons were also made between groups using ANOVA in which the factors were animal, group (control or *fat-1*) and inhibitor concentration. All statistical analysis was performed using JMP software (SAS Institute, Cary, NC). All results are presented as mean ± SEM and statistical significance is reported at *P*≤0.05.

## Supporting Information

Table S1 Phospholipid composition of liver mitochondria from *fat-1* and control mice.
(0.03 MB DOC)

Table S2 Fatty acid composition of phosphatidylcholine from liver mitochondria of control and *fat-1* mice.
(0.05 MB DOC)

Table S3 Fatty acid composition of phosphatidylethanolamine from liver mitochondria of control and *fat-1* mice.
(0.05 MB DOC)

Table S4 Fatty acid composition of cardiolipin from liver mitochondria of control and *fat-1* mice.
(0.05 MB DOC)

Table S5 Fatty acid composition of phosphatidylserine from liver mitochondria of control and *fat-1* mice.
(0.05 MB DOC)

Table S6 Fatty acid composition of lysophosphatidylcholine from liver mitochondria of control and *fat-1* mice.
(0.05 MB DOC)

Figure S1 The rate of palmitate oxidation as a measure of β-oxidation in liver mitochondria from control and *fat-1* mice. Mitochondria were incubated with [14C] palmitic acid, as described in the text. There were no differences (P>0.05) between groups.
(6.31 MB TIF)

## References

[1] Breslow JL (2006). n-3 fatty acids and cardiovascular disease.. Am J Clin Nutr.

[2] Mozaffarian D, Rimm EB (2006). Fish intake, contaminants, and human health: evaluating the risks and the benefits.. JAMA.

[3] Grimm H, Mayer K, Mayser P, Eigenbrodt E (2002). Regulatory potential of n-3 fatty acids in immunological and inflammatory processes.. Br J Nutr.

[4] Parker G, Gibson NA, Brotchie H, Heruc G, Rees AM (2006). Omega-3 fatty acids and mood disorders.. Am J Psychiatry.

[5] Kubo K, Saito M, Tadokoro T, Maekawa A (1997). Changes in susceptibility of tissues to lipid peroxidation after ingestion of various levels of docosahexaenoic acid and vitamin E.. Br J Nutr.

[6] Valencak TG, Ruf T (2007). N-3 polyunsaturated fatty acids impair lifespan but have no role for metabolism.. Aging Cell.

[7] Pamplona R, Barja G, Portero-Otin M (2002). Membrane fatty acid unsaturation, protection against oxidative stress, and maximum life span: a homeoviscous-longevity adaptation?. Ann N Y Acad Sci.

[8] Pamplona R, Portero-Otin M, Riba D, Ruiz C, Prat J (1998). Mitochondrial membrane peroxidizability index is inversely related to maximum life span in mammals.. J Lipid Res.

[9] Hulbert AJ (2005). On the importance of fatty acid composition of membranes for aging.. J Theor Biol.

[10] Murphy MP (2009). How mitochondria produce reactive oxygen species.. Biochem J.

[11] Daum G (1985). Lipids of mitochondria.. Biochim Biophys Acta.

[12] Fry M, Green DE (1981). Cardiolipin requirement for electron transfer in complex I and III of the mitochondrial respiratory chain.. J Biol Chem.

[13] Gomez B, Robinson NC (1999). Phospholipase digestion of bound cardiolipin reversibly inactivates bovine cytochrome bc1.. Biochemistry.

[14] Fry M, Green DE (1980). Cardiolipin requirement by cytochrome oxidase and the catalytic role of phospholipid.. Biochem Biophys Res Commun.

[15] Sedlak E, Robinson NC (1999). Phospholipase A(2) digestion of cardiolipin bound to bovine cytochrome c oxidase alters both activity and quaternary structure.. Biochemistry.

[16] Barzanti V, Battino M, Baracca A, Cavazzoni M, Cocchi M (1994). The effect of dietary lipid changes on the fatty acid composition and function of liver, heart and brain mitochondria in the rat at different ages.. Br J Nutr.

[17] Ramsey JJ, Harper ME, Humble SJ, Koomson EK, Ram JJ (2005). Influence of mitochondrial membrane fatty acid composition on proton leak and H2O2 production in liver.. Comp Biochem Physiol B Biochem Mol Biol.

[18] Hong MY, Chapkin RS, Barhoumi R, Burghardt RC, Turner ND (2002). Fish oil increases mitochondrial phospholipid unsaturation, upregulating reactive oxygen species and apoptosis in rat colonocytes.. Carcinogenesis.

[19] Watkins SM, Carter LC, German JB (1998). Docosahexaenoic acid accumulates in cardiolipin and enhances HT-29 cell oxidant production.. J Lipid Res.

[20] Kang JX, Wang J, Wu L, Kang ZB (2004). Transgenic mice: fat-1 mice convert n-6 to n-3 fatty acids.. Nature.

[21] Spychalla JP, Kinney AJ, Browse J (1997). Identification of an animal omega-3 fatty acid desaturase by heterologous expression in Arabidopsis.. Proc Natl Acad Sci U S A.

[22] Benard G, Faustin B, Passerieux E, Galinier A, Rocher C (2006). Physiological diversity of mitochondrial oxidative phosphorylation.. Am J Physiol Cell Physiol.

[23] Lenaz G, Genova ML (2009). Structural and functional organization of the mitochondrial respiratory chain: a dynamic super-assembly.. Int J Biochem Cell Biol.

[24] Bhattacharya A, Chandrasekar B, Rahman MM, Banu J, Kang JX (2006). Inhibition of inflammatory response in transgenic fat-1 mice on a calorie-restricted diet.. Biochem Biophys Res Commun.

[25] Kang JX (2005). From fat to fat-1: a tale of omega-3 fatty acids.. J Membr Biol.

[26] Schmocker C, Weylandt KH, Kahlke L, Wang J, Lobeck H (2007). Omega-3 fatty acids alleviate chemically induced acute hepatitis by suppression of cytokines.. Hepatology.

[27] Berger A, German JB, Gershwin ME (1993). Biochemistry of cardiolipin: sensitivity to dietary fatty acids.. Adv Food Nutr Res.

[28] Swanson JE, Kinsella JE (1986). Dietary n-3 polyunsaturated fatty acids: modification of rat cardiac lipids and fatty acid composition.. J Nutr.

[29] Yamaoka S, Urade R, Kito M (1988). Mitochondrial function in rats is affected by modification of membrane phospholipids with dietary sardine oil.. J Nutr.

[30] Hoch FL (1992). Cardiolipins and biomembrane function.. Biochim Biophys Acta.

[31] McMillin JB, Bick RJ, Benedict CR (1992). Influence of dietary fish oil on mitochondrial function and response to ischemia.. Am J Physiol.

[32] Lemieux H, Blier PU, Tardif JC (2008). Does membrane fatty acid composition modulate mitochondrial functions and their thermal sensitivities?. Comp Biochem Physiol A Mol Integr Physiol.

[33] Stillwell W, Jenski LJ, Crump FT, Ehringer W (1997). Effect of docosahexaenoic acid on mouse mitochondrial membrane properties.. Lipids.

[34] Demaison L, Sergiel JP, Moreau D, Grynberg A (1994). Influence of the phospholipid n-6/n-3 polyunsaturated fatty acid ratio on the mitochondrial oxidative metabolism before and after myocardial ischemia.. Biochim Biophys Acta.

[35] Malis CD, Weber PC, Leaf A, Bonventre JV (1990). Incorporation of marine lipids into mitochondrial membranes increases susceptibility to damage by calcium and reactive oxygen species: evidence for enhanced activation of phospholipase A2 in mitochondria enriched with n-3 fatty acids.. Proc Natl Acad Sci U S A.

[36] Rossignol R, Faustin B, Rocher C, Malgat M, Mazat JP (2003). Mitochondrial threshold effects.. Biochem J.

[37] Cortes-Rojo C, Calderon-Cortes E, Clemente-Guerrero M, Estrada-Villagomez M, Manzo-Avalos S (2009). Elucidation of the effects of lipoperoxidation on the mitochondrial electron transport chain using yeast mitochondria with manipulated fatty acid content.. J Bioenerg Biomembr.

[38] Martinez M, Hernandez AI, Martinez N, Ferrandiz ML (1996). Age-related increase in oxidized proteins in mouse synaptic mitochondria.. Brain Res.

[39] Parise G, Brose AN, Tarnopolsky MA (2005). Resistance exercise training decreases oxidative damage to DNA and increases cytochrome oxidase activity in older adults.. Exp Gerontol.

[40] Herrero A, Portero-Otin M, Bellmunt MJ, Pamplona R, Barja G (2001). Effect of the degree of fatty acid unsaturation of rat heart mitochondria on their rates of H2O2 production and lipid and protein oxidative damage.. Mech Ageing Dev.

[41] Lopez-Torres M, Gredilla R, Sanz A, Barja G (2002). Influence of aging and long-term caloric restriction on oxygen radical generation and oxidative DNA damage in rat liver mitochondria.. Free Radic Biol Med.

[42] Hoffman DL, Brookes PS (2009). Oxygen sensitivity of mitochondrial reactive oxygen species generation depends on metabolic conditions.. J Biol Chem.

[43] Lambert AJ, Brand MD (2004). Inhibitors of the quinone-binding site allow rapid superoxide production from mitochondrial NADH:ubiquinone oxidoreductase (complex I).. J Biol Chem.

[44] Porter RK, Hulbert AJ, Brand MD (1996). Allometry of mitochondrial proton leak: influence of membrane surface area and fatty acid composition.. Am J Physiol.

[45] Clarke SD, Jump DB (1994). Dietary polyunsaturated fatty acid regulation of gene transcription.. Annu Rev Nutr.

[46] Price PT, Nelson CM, Clarke SD (2000). Omega-3 polyunsaturated fatty acid regulation of gene expression.. Curr Opin Lipidol.

[47] Flachs P, Horakova O, Brauner P, Rossmeisl M, Pecina P (2005). Polyunsaturated fatty acids of marine origin upregulate mitochondrial biogenesis and induce beta-oxidation in white fat.. Diabetologia.

[48] Rahman M, Halade GV, Bhattacharya A, Fernandes G (2009). The fat-1 transgene in mice increases antioxidant potential, reduces pro-inflammatory cytokine levels, and enhances PPAR γαμμα and SIRT-1 expression on a calorie restricted diet.. Oxidative Medicine and Cellular Longevity.

[49] Venditti P, De Rosa R, Di Meo S (2004). Effect of cold-induced hyperthyroidism on H2O2 production and susceptibility to stress conditions of rat liver mitochondria.. Free Radic Biol Med.

[50] Arvier M, Lagoutte L, Johnson G, Dumas JF, Sion B (2007). Adenine nucleotide translocator promotes oxidative phosphorylation and mild uncoupling in mitochondria after dexamethasone treatment.. Am J Physiol Endocrinol Metab.

[51] Barrientos A (2002). In vivo and in organello assessment of OXPHOS activities.. Methods.

[52] Birch-Machin M, Jackson S, Kler RS, Turnbull DM (1993). Study of skeletal muscle mitochondrial dysfunction.. Methods in Toxicology.

[53] Birch-Machin MA, Briggs HL, Saborido AA, Bindoff LA, Turnbull DM (1994). An evaluation of the measurement of the activities of complexes I-IV in the respiratory chain of human skeletal muscle mitochondria.. Biochem Med Metab Biol.

[54] Kwong LK, Sohal RS (2000). Age-related changes in activities of mitochondrial electron transport complexes in various tissues of the mouse.. Arch Biochem Biophys.

[55] Trounce IA, Kim YL, Jun AS, Wallace DC (1996). Assessment of mitochondrial oxidative phosphorylation in patient muscle biopsies, lymphoblasts, and transmitochondrial cell lines.. Methods Enzymol.

[56] Ramsey JJ, Hagopian K, Kenny TM, Koomson EK, Bevilacqua L (2004). Proton leak and hydrogen peroxide production in liver mitochondria from energy-restricted rats.. Am J Physiol Endocrinol Metab.

[57] Brand MD, Brown GC, Cooper CE (1995). Measurement of mitochondrial protonmotive force.. Bioenergetics.

[58] Toroser D, Orr WC, Sohal RS (2007). Carbonylation of mitochondrial proteins in Drosophila melanogaster during aging.. Biochem Biophys Res Commun.

[59] Caro P, Gomez J, Lopez-Torres M, Sanchez I, Naudi A (2008). Effect of every other day feeding on mitochondrial free radical production and oxidative stress in mouse liver.. Rejuvenation Res.

[60] Caro P, Gomez J, Sanchez I, Garcia R, Lopez-Torres M (2008). Effect of 40% restriction of dietary amino acids (except methionine) on mitochondrial oxidative stress and biogenesis, AIF and SIRT1 in rat liver..

[61] Hyslop PA, Sklar LA (1984). A quantitative fluorimetric assay for the determination of oxidant production by polymorphonuclear leukocytes: its use in the simultaneous fluorimetric assay of cellular activation processes.. Anal Biochem.

[62] Folch J, Lees M, Sloane Stanley GH (1957). A simple method for the isolation and purification of total lipides from animal tissues.. J Biol Chem.

[63] Watkins SM, Lin TY, Davis RM, Ching JR, DePeters EJ (2001). Unique phospholipid metabolism in mouse heart in response to dietary docosahexaenoic or alpha-linolenic acids.. Lipids.

[64] Fernandez-Ayala DJ, Lopez-Lluch G, Garcia-Valdes M, Arroyo A, Navas P (2005). Specificity of coenzyme Q10 for a balanced function of respiratory chain and endogenous ubiquinone biosynthesis in human cells.. Biochim Biophys Acta.

[65] Arroyo A, Navarro F, Navas P, Villalba JM (1998). Ubiquinol regeneration by plasma membrane ubiquinone reductase.. Protoplasma.

[66] Schultz JR, Ellerby LM, Gralla EB, Valentine JS, Clarke CF (1996). Autoxidation of ubiquinol-6 is independent of superoxide dismutase.. Biochemistry.

[67] Gerard-Monnier D, Erdelmeier I, Regnard K, Moze-Henry N, Yadan JC (1998). Reactions of 1-methyl-2-phenylindole with malondialdehyde and 4-hydroxyalkenals. Analytical applications to a colorimetric assay of lipid peroxidation.. Chem Res Toxicol.

[68] Murase T, Aoki M, Wakisaka T, Hase T, Tokimitsu I (2002). Anti-obesity effect of dietary diacylglycerol in C57BL/6J mice: dietary diacylglycerol stimulates intestinal lipid metabolism.. J Lipid Res.

