# Supplementary material for: Complex I-Associated Hydrogen Peroxide Production Is Decreased and Electron Transport Chain Enzyme Activities Are Altered in n-3 Enriched fat-1 Mice
Source: PLoS One. 2010 Sep 13;5(9):e12696. doi: 10.1371/journal.pone.0012696 (PMC2938348; doi:10.1371/journal.pone.0012696)
Supplement: Table S1 — Phospholipid composition of liver mitochondria from fat-1 and control mice. (0.03 MB DOC) [file pone.0012696.s001.doc]

**Table S1.** Phospholipid composition of liver mitochondria from *fat-1* and control mice.

| **Phospholipid Class** | **Control (% of total)** | ***fat-1* (% of total)** |
| --- | --- | --- |
| Phosphatidylcholine | 43.7 ± 4.3 | 40.8 ± 3.4 |
| Phosphatidylethanolamine | 31.2 ± 3.3 | 30.3 ± 2.3 |
| Cardiolipin | 21.4 ± 2.1 | 23.8 ± 2.7 |
| Phosphatidylserine | 3.2 ± 0.3 | 4.3 ± 0.7 |
| Lysophosphatidylcholine | 0.54 ± 0.04 | 0.75 ± 0.03 |

There were no significant differences (*P* > 0.10) between control and *fat-1* groups for any of the phospholipids.
