# Supplementary material for: Complex I-Associated Hydrogen Peroxide Production Is Decreased and Electron Transport Chain Enzyme Activities Are Altered in n-3 Enriched fat-1 Mice
Source: PLoS One. 2010 Sep 13;5(9):e12696. doi: 10.1371/journal.pone.0012696 (PMC2938348; doi:10.1371/journal.pone.0012696)
Supplement: Table S2 — Fatty acid composition of phosphatidylcholine from liver mitochondria of control and fat-1 mice. (0.05 MB DOC) [file pone.0012696.s002.doc]

**Table S2.** Fatty acid composition of phosphatidylcholine from liver mitochondria of control and *fat-1* mice.

| **Fatty Acids** | **Control (% of total)** | ***fat-1* (% of total)** |
| --- | --- | --- |
| 14:0 | 0.09 ± 0.01 | 0.13 ± 0.03 |
| 15:0 | 0.15 ± 0.01 | 0.17 ± 0.04 |
| 16:0 | 28.96 ± 0.38 | 29.34 ± 0.65 |
| 18:0 | 11.11 ± 0.30 | 9.85 ± 0.34^*^ |
| 20:0 | 0.24 ± 0.02 | 0.22 ± 0.02 |
| 22:0 | 0.011 ± 0.004 | 0.018 ± 0.005 |
| 24:0 | 0.023 ± 0.005 | 0.020 ± 0.005 |
| 14:1n5 | 0.0026 ± 0.0002 | 0.007 ± 0.003 |
| 16:1n7 | 1.12 ± 0.10 | 1.38 ± 0.13 |
| 18:1n7 | 1.65 ± 0.14 | 2.54 ± 0.35^*^ |
| 18:1n9 | 8.16 ± 0.33 | 9.67 ± 0.16^*^ |
| 20:1n9 | 0.18 ± 0.01 | 0.22 ± 0.02 |
| 20:3n9 | 0.07 ± 0.01 | 0.15 ± 0.02^*^ |
| 22:1n9 | 0.011 ± 0.002 | 0.022 ± 0.010 |
| 24:1n9 | 0.008 ± 0.002 | 0.008 ± 0.001 |
| 18:2n6 | 17.31 ± 0.33 | 18.38 ± 0.37 |
| 18:3n6 | 0.30 ± 0.01 | 0.27 ± 0.02 |
| 20:2n6 | 0.25 ± 0.02 | 0.37 ± 0.02^*^ |
| 20:3n6 | 2.50 ± 0.13 | 2.41 ± 0.18 |
| 20:4n6 | 12.65 ± 0.52 | 6.30 ± 0.35^*^ |
| 22:2n6 | 0.008 ± 0.001 | 0.019 ± 0.002^*^ |
| 22:4n6 | 0.074 ± 0.012 | 0.054 ± 0.003 |
| 22:5n6 | 0.070 ± 0.009 | 0.063 ± 0.003 |
| 18:3n3 | 0.10 ± 0.02 | 0.15 ± 0.02 |
| 18:4n3 | 0.004 ± 0.001 | 0.007 ± 0.001^*^ |
| 20:4n3 | 0.037 ± 0.003 | 0.205 ± 0.024^*^ |
| 20:5n3 | 1.06 ± 0.10 | 2.63 ± 0.31^*^ |
| 22:5n3 | 0.54 ± 0.04 | 0.82 ± 0.03^*^ |
| 22:6n3 | 13.29 ± 0.16 | 14.56 ± 0.42^*^ |

All values are expressed as a percent of total fatty acids.

^*^Indicates a significant difference (*P* < 0.05) between control and *fat-1* groups.

Dimethoxyacetyl and trans fats have been excluded from the table because levels of these fatty acids were negligible in both control and *fat-1* mice.
