# Supplementary material for: Complex I-Associated Hydrogen Peroxide Production Is Decreased and Electron Transport Chain Enzyme Activities Are Altered in n-3 Enriched fat-1 Mice
Source: PLoS One. 2010 Sep 13;5(9):e12696. doi: 10.1371/journal.pone.0012696 (PMC2938348; doi:10.1371/journal.pone.0012696)
Supplement: Table S3 — Fatty acid composition of phosphatidylethanolamine from liver mitochondria of control and fat-1 mice. (0.05 MB DOC) [file pone.0012696.s003.doc]

**Table S3.** Fatty acid composition of phosphatidylethanolamine from liver mitochondria of control and *fat-1* mice.

| **Fatty Acids** | **Control (% of total)** | ***fat-1* (% of total)** |
| --- | --- | --- |
| 14:0 | 0.04 ± 0.01 | 0.07 ± 0.02 |
| 15:0 | 0.047 ± 0.004 | 0.054 ± 0.013 |
| 16:0 | 15.21 ± 0.26 | 16.16 ± 0.20^*^ |
| 18:0 | 24.19 ± 0.41 | 23.09 ± 0.57 |
| 20:0 | 0.33 ± 0.02 | 0.31 ± 0.04 |
| 22:0 | 0.016 ± 0.008 | 0.007 ± 0.004 |
| 24:0 | 0.010 ± 0.004 | 0.013 ± 0.007 |
| 14:1n5 | 0.008 ± 0.002 | 0.022 ± 0.001 |
| 16:1n7 | 0.54 ± 0.05 | 0.65 ± 0.06 |
| 18:1n7 | 0.95 ± 0.10 | 1.43 ± 0.21 |
| 18:1n9 | 6.38 ± 0.15 | 6.62 ± 0.34 |
| 20:1n9 | 0.27 ± 0.01 | 0.28 ± 0.01 |
| 20:3n9 | 0.16 ± 0.02 | 0.37 ± 0.04^*^ |
| 22:1n9 | 0.016 ± 0.006 | 0.012 ± 0.002 |
| 24:1n9 | 0.019 ± 0.010 | 0.008 ± 0.002 |
| 18:2n6 | 5.20 ± 0.10 | 5.74 ± 0.25 |
| 18:3n6 | 0.059 ± 0.002 | 0.052 ± 0.005 |
| 20:2n6 | 0.11 ± 0.02 | 0.13 ± 0.01^*^ |
| 20:3n6 | 1.02 ± 0.04 | 1.29 ± 0.08^*^ |
| 20:4n6 | 26.13 ± 0.37 | 19.86 ± 0.48^*^ |
| 22:2n6 | 0.0075 ± 0.0003 | 0.010 ± 0.003 |
| 22:4n6 | 0.101 ± 0.004 | 0.081 ± 0.003^*^ |
| 22:5n6 | 0.078 ± 0.007 | 0.080 ± 0.005 |
| 18:3n3 | 0.053 ± 0.002 | 0.081 ± 0.012 |
| 18:4n3 | 0.012 ± 0.001 | 0.014 ± 0.003 |
| 20:4n3 | 0.022 ± 0.001 | 0.073 ± 0.007^*^ |
| 20:5n3 | 1.33 ± 0.11 | 3.85 ± 0.39^*^ |
| 22:5n3 | 0.63 ± 0.03 | 0.94 ± 0.042^*^ |
| 22:6n3 | 16.70 ± 0.31 | 18.44 ± 0.44^*^ |

All values are expressed as a percent of total fatty acids.

^*^Indicates a significant difference (*P* < 0.05) between control and *fat-1* groups.

Dimethoxyacetyl and trans fats have been excluded from the table because levels of these fatty acids were negligible in both control and *fat-1* mice.
