# Supplementary material for: Complex I-Associated Hydrogen Peroxide Production Is Decreased and Electron Transport Chain Enzyme Activities Are Altered in n-3 Enriched fat-1 Mice
Source: PLoS One. 2010 Sep 13;5(9):e12696. doi: 10.1371/journal.pone.0012696 (PMC2938348; doi:10.1371/journal.pone.0012696)
Supplement: Table S4 — Fatty acid composition of cardiolipin from liver mitochondria of control and fat-1 mice. (0.05 MB DOC) [file pone.0012696.s004.doc]

**Table S4.** Fatty acid composition of cardiolipin from liver mitochondria of control and *fat-1* mice.

| **Fatty Acids** | **Control (% of total)** | ***fat-1* (% of total)** |
| --- | --- | --- |
| 14:0 | 0.40 ± 0.03 | 0.40 ± 0.03 |
| 15:0 | 0.120 ± 0.006 | 0.147 ± 0.039 |
| 16:0 | 8.75 ± 0.93 | 7.74 ± 0.57 |
| 18:0 | 4.05 ± 0.35 | 3.44 ± 0.25 |
| 20:0 | 0.12 ± 0.01 | 0.11 ± 0.01 |
| 22:0 | 0.101 ± 0.006 | 0.077 ± 0.006^*^ |
| 24:0 | 0.054 ± 0.008 | 0.046 ± 0.006 |
| 14:1n5 | 0.019 ± 0.005 | 0.025 ± 0.005 |
| 16:1n7 | 2.57 ± 0.18 | 3.07 ± 0.47 |
| 18:1n7 | 5.92 ± 0.19 | 5.32 ± 0.26 |
| 18:1n9 | 8.11 ± 0.35 | 9.01 ± 0.76 |
| 20:1n9 | 0.31 ± 0.01 | 0.28 ± 0.04 |
| 20:3n9 | 0.07 ± 0.02 | 0.06 ± 0.01 |
| 22:1n9 | 2.43 ± 0.36 | 2.15 ± 0.33 |
| 24:1n9 | 0.047 ± 0.012 | 0.067 ± 0.012 |
| 18:2n6 | 53.69 ± 1.41 | 55.03 ± 1.93 |
| 18:3n6 | 0.127 ± 0.003 | 0.100 ± 0.005^*^ |
| 20:2n6 | 0.84 ± 0.09 | 0.65 ± 0.16 |
| 20:3n6 | 1.93 ± 0.08 | 1.89 ± 0.26 |
| 20:4n6 | 3.72 ± 0.36 | 2.31 ± 0.17^*^ |
| 22:2n6 | 0.049 ± 0.008 | 0.048 ± 0.009 |
| 22:4n6 | 0.071 ± 0.019 | 0.049 ± 0.010 |
| 22:5n6 | 0.062 ± 0.012 | 0.039 ± 0.010 |
| 18:3n3 | 0.265 ± 0.010 | 0.373 ± 0.020^*^ |
| 18:4n3 | 0.038 ± 0.008 | 0.037 ± 0.005 |
| 20:4n3 | 0.072 ± 0.005 | 0.242 ± 0.039^*^ |
| 20:5n3 | 0.33 ± 0.04 | 0.60 ± 0.05^*^ |
| 22:5n3 | 0.40 ± 0.02 | 0.51 ± 0.06 |
| 22:6n3 | 6.38 ± 0.03 | 6.08 ± 0.65 |

All values are expressed as a percent of total fatty acids.

^*^Indicates a significant difference (*P* < 0.05) between control and *fat-1* groups.

Dimethoxyacetyl and trans fats have been excluded from the table because levels of these fatty acids were negligible in both control and *fat-1* mice.
