# Supplementary material for: Complex I-Associated Hydrogen Peroxide Production Is Decreased and Electron Transport Chain Enzyme Activities Are Altered in n-3 Enriched fat-1 Mice
Source: PLoS One. 2010 Sep 13;5(9):e12696. doi: 10.1371/journal.pone.0012696 (PMC2938348; doi:10.1371/journal.pone.0012696)
Supplement: Table S5 — Fatty acid composition of phosphatidylserine from liver mitochondria of control and fat-1 mice. (0.05 MB DOC) [file pone.0012696.s005.doc]

**Table S5.** Fatty acid composition of phosphatidylserine from liver mitochondria of control and *fat-1* mice.

| **Fatty Acids** | **Control (% of total)** | ***fat-1* (% of total)** |
| --- | --- | --- |
| 14:0 | 0.60 ± 0.08 | 0.96 ± 0.14 |
| 15:0 | 0.258 ± 0.039 | 0.303 ± 0.059 |
| 16:0 | 14.50 ± 0.53 | 18.30 ± 0.92^*^ |
| 18:0 | 33.67 ± 0.43 | 28.85 ± 1.85^*^ |
| 20:0 | 0.57 ± 0.04 | 0.48 ± 0.05 |
| 22:0 | 0.264 ± 0.050 | 0.208 ± 0.030 |
| 24:0 | 0.206 ± 0.054 | 0.183 ± 0.025 |
| 14:1n5 | 0.069 ± 0.012 | 0.092 ± 0.016 |
| 16:1n7 | 0.34 ± 0.02 | 0.83 ± 0.15^*^ |
| 18:1n7 | 0.76 ± 0.06 | 1.50 ± 0.20^*^ |
| 18:1n9 | 3.83 ± 0.19 | 9.26 ± 2.83 |
| 20:1n9 | 0.21 ± 0.02 | 0.36 ± 0.04^*^ |
| 20:3n9 | 0 | 0 |
| 22:1n9 | 0.10 ± 0.03 | 0.09 ± 0.03 |
| 24:1n9 | 0.113 ± 0.018 | 0.132 ± 0.020 |
| 18:2n6 | 3.03 ± 0.31 | 4.65 ± 1.02 |
| 18:3n6 | 0 | 0 |
| 20:2n6 | 0.13 ± 0.01 | 0.18 ± 0.04 |
| 20:3n6 | 0.68 ± 0.04 | 0.61 ± 0.05 |
| 20:4n6 | 16.26 ± 0.36 | 9.00 ± 1.24^*^ |
| 22:2n6 | 0 | 0 |
| 22:4n6 | 0.212 ± 0.011 | 0.108 ± 0.012^*^ |
| 22:5n6 | 0.324 ± 0.034 | 0.214 ± 0.046 |
| 18:3n3 | 0.124 ± 0.032 | 0.151 ± 0.037 |
| 18:4n3 | 0.227 ± 0.017 | 0.022 ± 0.004^*^ |
| 20:4n3 | 0 | 0 |
| 20:5n3 | 1.03 ± 0.10 | 2.22 ± 0.40^*^ |
| 22:5n3 | 0.70 ± 0.05 | 0.95 ± 0.12 |
| 22:6n3 | 21.70 ± 0.75 | 20.18 ± 1.96 |

All values are expressed as a percent of total fatty acids.

^*^Indicates a significant difference (*P* < 0.05) between control and *fat-1* groups.

Dimethoxyacetyl and trans fats have been excluded from the table because levels of these fatty acids were negligible in both control and *fat-1* mice.
