# Supplementary material for: Complex I-Associated Hydrogen Peroxide Production Is Decreased and Electron Transport Chain Enzyme Activities Are Altered in n-3 Enriched fat-1 Mice
Source: PLoS One. 2010 Sep 13;5(9):e12696. doi: 10.1371/journal.pone.0012696 (PMC2938348; doi:10.1371/journal.pone.0012696)
Supplement: Table S6 — Fatty acid composition of lysophosphatidylcholine from liver mitochondria of control and fat-1 mice. (0.05 MB DOC) [file pone.0012696.s006.doc]

**Table S6.** Fatty acid composition of lysophosphatidylcholine from liver mitochondria of control and *fat-1* mice.

| **Fatty Acids** | **Control (% of total)** | ***fat-1* (% of total)** |
| --- | --- | --- |
| 14:0 | 1.62 ± 0.15 | 1.61 ± 0.34 |
| 15:0 | 0.75 ± 0.11 | 0.87 ± 0.25 |
| 16:0 | 35.03 ± 0.59 | 35.32 ± 0.88 |
| 18:0 | 18.54 ± 0.73 | 19.90 ± 1.29 |
| 20:0 | 0.71 ± 0.06 | 0.75 ± 0.09 |
| 22:0 | 0.25 ± 0.02 | 0.29 ± 0.07 |
| 24:0 | 0.28 ± 0.03 | 0.51 ± 0.20 |
| 14:1n5 | 0.17 ± 0.03 | 0.19 ± 0.05 |
| 16:1n7 | 1.71 ± 0.28 | 1.30 ± 0.10 |
| 18:1n7 | 1.53 ± 0.85 | 2.00 ± 0.26 |
| 18:1n9 | 7.21 ± 0.24 | 8.05 ± 0.27 |
| 20:1n9 | 0.28 ± 0.02 | 0.34 ± 0.06 |
| 20:3n9 | 0.055 ± 0.009 | 0.090 ± 0.019 |
| 22:1n9 | 0.069 ± 0.018 | 0.181 ± 0.041 |
| 24:1n9 | 0.119 ± 0.004 | 0.154 ± 0.032 |
| 18:2n6 | 13.58 ± 0.68 | 11.85 ± 1.44 |
| 18:3n6 | 0.27 ± 0.03 | 0.26 ± 0.08 |
| 20:2n6 | 0.19 ± 0.01 | 0.27 ± 0.02^*^ |
| 20:3n6 | 1.27 ± 0.08 | 1.15 ± 0.12 |
| 20:4n6 | 7.56 ± 0.25 | 4.62 ± 0.32^*^ |
| 22:2n6 | 0 | 0 |
| 22:4n6 | 0.23 ± 0.13 | 0.10 ± 0.04 |
| 22:5n6 | 0.11 ± 0.01 | 0.19 ± 0.06 |
| 18:3n3 | 0.27 ± 0.10 | 0.22 ± 0.03 |
| 18:4n3 | 0 | 0 |
| 20:4n3 | 0.050 ± 0.003 | 0.118 ± 0.021 |
| 20:5n3 | 0.46 ± 0.04 | 1.00 ± 0.14^*^ |
| 22:5n3 | 0.32 ± 0.03 | 0.50 ± 0.04^*^ |
| 22:6n3 | 7.33 ± 0.39 | 8.17 ± 0.40 |

All values are expressed as a percent of total fatty acids.

^*^Indicates a significant difference (*P* < 0.05) between control and *fat-1* groups.

Dimethoxyacetyl and trans fats have been excluded from the table because levels of these fatty acids were negligible in both control and *fat-1* mice.
