# Supplementary figures and images for: Complex I-Associated Hydrogen Peroxide Production Is Decreased and Electron Transport Chain Enzyme Activities Are Altered in n-3 Enriched fat-1 Mice
Source: PLoS One. 2010 Sep 13;5(9):e12696. doi: 10.1371/journal.pone.0012696 (PMC2938348; doi:10.1371/journal.pone.0012696)

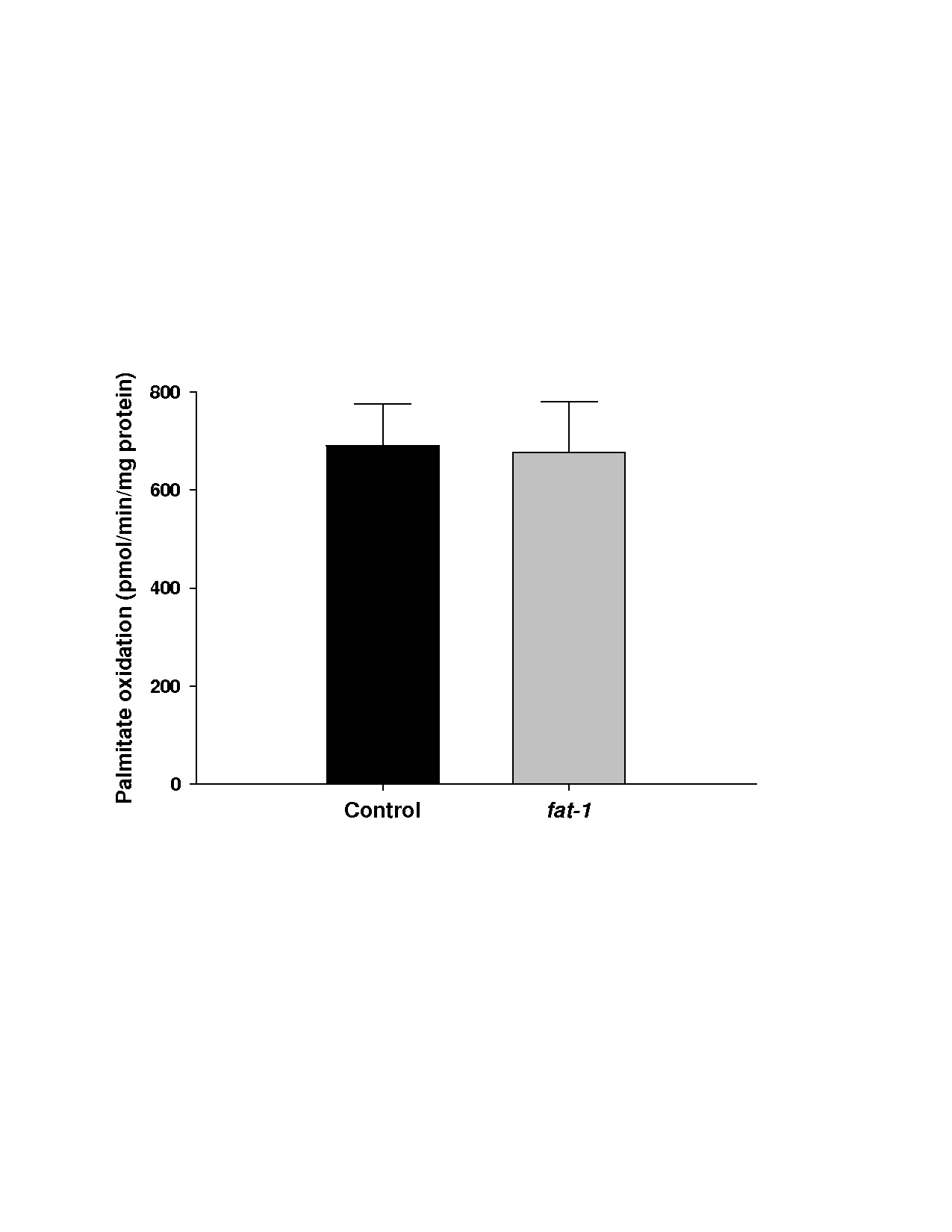

Supplement: Figure S1 — The rate of palmitate oxidation as a measure of β-oxidation in liver mitochondria from control and fat-1 mice. Mitochondria were incubated with [14C] palmitic acid, as described in the text. There were no differences (P>0.05) between groups. (6.31 MB TIF) [file pone.0012696.s007.tif]
